# Scientometric Analysis and Mapping of Scientific Articles on Diabetic Retinopathy

**Published:** 2015

**Authors:** Shahrokh RAMIN, Reza GHAREBAGHI, Fatemeh HEIDARY

**Affiliations:** 1 Ophthalmic Research Center, Shahid Beheshti University of Medical Sciences, Tehran, Iran; 2 Health Policy Research Center, Shiraz University of Medical Sciences, Shiraz, Iran

**Keywords:** Diabetic Retinopathy, Bibliometrics, Historiography, Scientometry, Citation Analysis

## Abstract

Diabetic retinopathy (DR) is the major cause of blindness among the working-age population globally. No systematic research has been previously performed to analyze the research published on DR, despite the need for it. This study aimed to analyze the scientific production on DR to draw overall roadmap of future research strategic planning in this field. A bibliometric method was used to obtain a view on the scientific production about DR by the data extracted from the Institute for Scientific Information (ISI). Articles about DR published in 1993–2013 were analyzed to obtain a view of the topic’s structure, history, and to document relationships. The trends in the most influential publications and authors were analyzed. Most highly cited articles addressed epidemiologic and translational research topics in this field. During the past 3 years, there has been a trend toward biomarker discovery and more molecular translational research. Areas such as gene therapy and micro-RNAs are also among the recent hot topics. Through analyzing the characteristics of papers and the trends in scientific production, we performed the first scientometric report on DR. Most influential articles have addressed epidemiology and translational research subjects in this field, which reflects that globally, the earlier diagnosis and treatment of this devastating disease still has the highest global priority.

## INTRODUCTION

An estimated 382 million people had diabetes in 2013; this is expected to rise to 592 million by 2035 (1). Diabetic retinopathy (DR), age-related macular degeneration (ARMD), glaucoma, and childhood causes are the most common causes of low vision in all countries (2). Estimates of the prevalence of DR vary by study and rates range from 17.6% in a study in India to 33.2% in a large United States study (3, 4). There were 126.6 million people with DR worldwide in 2010; this is expected to increase to 191.0 million by 2030. The number of patients with vision-threatening DR will increase from 37.3 million to 56.3 million (5, 6). If a diabetic patient does not have retinopathy, studies suggest that the risk of developing new retinopathy ranges between 5% and 10% annually. Thus, there is an urgent need for prompt action.

Preventing and treating DR are major concerns in this field. If fundamental social and political changes are available, the prevention of diabetes would be the best approach to prevent DR. Factors which lower the risk of visual morbidities and disease progression in diabetic patients include optimal blood glucose and blood pressure control beside regular ocular examinations and prompt laser treatment of macular edema as well as proliferative retinopathy. The Wisconsin Epidemiologic Study of Diabetic Retinopathy (i.e., WESDR) first identified key risk factors for DR such as longer duration of diabetes, hyperglycemia, and hypertension (7, 8). Based on the finding of this study and other studies, new screening strategies need to be developed that detect potential vision-threatening retinopathy early in clinical and nonclinical settings. Genetic risk factors for diabetes and DR should be identified, and the interactions between genes and metabolic control should be examined; these factors will help in risk stratification and in preventing vision loss (9). Therefore, implementing of novel, feasible, and sustainable strategies to control the growing current of DR is a significant challenge. Part of the challenge is the need for global level research strategic planning for preventing and treating DR.

Many clinical reviews and meta-analyses exist on DR, and scientometric studies investigating other topics in ophthalmology exist; however, quantitative description of publications specifically on DR is lacking. Recent bibliometric analysis of scientific publications has been performed for individual and institutional output analysis, and for assessing the scientific advancements and motivations of researchers and identifying current research directions in a specific field; fund assignments and subsequent research designs can be enhanced using such data because it will predict how this field will move forward (10, 11). Mapping the external and internal features of a scientific field by tracing the core production or citations, would aid in research that is more global strategic planning. Thus, we aimed to analyze the scientific productions on DR to define a general roadmap for future research strategic planning in this field.

## MATERIAL AND METHODS

**Data Source**

A descriptive bibliometric study of scientific papers about DR was conducted. For this purpose, the ISI Web of Science database (available at http://www.isiknowledge.com) was used because it is a major source for bibliometrics, citations, and other academic impact information of scientific articles in various branches of sciences. All three resources available in the ISI web of science were used for this purpose (Science Citation Index Expanded; Social Sciences Citation Index; the Arts & Humanities Citation Index, A&HCI.

**Search Strategies**

For the best keywords, we created a list from the Medical Subject Headings (MeSH), which is provided by the National Library of Medicine (NLM, Bethesda, MD, USA) to index the contents of PubMed. The adopted search strategy was Title: ((Diabet* and Retinopathy) or (Diabetic Retinopathy)) as the search keyword. This yielded 3228 publications. The ‘*’ is a wildcard that can take any value. Our search focused on articles published during 1993–2013. Our search was performed in Feb 2014. We included only research articles in the analysis and excluded meeting abstracts, case reports, review articles as well as letters.

**Data Analysis**

We retrieved documents related to main journals in this field, articles’ language, the publication year, first author, geographical distribution, institutional affiliations and citations of the paper by other papers from the ISI and analyzed with the analyze function provided by the ISI database. Also, we used the Journal Citation Reports (available at http://scientific.thomson.com/products/jcr) to derive journal’s impact factor. Software for statistical analysis in this study was Microsoft Excel 2003 computer spreadsheet software (Microsoft; Redmond, WA, USA). Analysis of related articles by HistCite software was performed considering the topic’s structure, history, and document relationships. We imported the bibliography derived from the web of science database to HistCite. Any articles that cited ≥ 100 were included in historiography of the DR research field from 1993 to 2013 (please refer to Appendix 1). Articles that were cited more than 100 times were evaluated by the country of affiliation of the first author and publishing journal. For identification of recent trends, the citation analysis was repeated for articles published from 2010 to 2013. For the citation analysis, two parameters were calculated: the local citation score (LCS) and the global citation score (GCS). The LCS lists all papers sorted by citation frequency within the local (i.e., the starting bibliography). By contrast, the GCS counts citations in the whole collection. For the citation burst analysis, the hundred keywords that generated the citation bursts were extracted, and then the nonspecific and general keywords were omitted.

**Figure 1:**
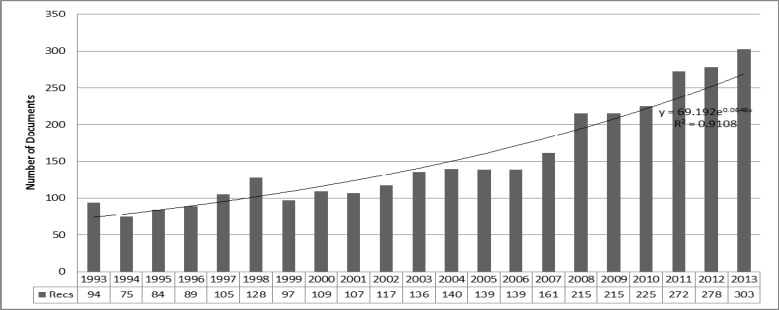
The Number of Papers Published Annually

The growth rate of 6.46% in the article numbers was observed.

## RESULTS

**Annual Publication Number During 1993–2013**

There were 3,228 research articles on DR in the ISI Web of Science published during 1993–2013. These papers were drafted by 11,591 authors, 2,771 institutions, and 93 countries. The articles were published in 547 journals in 10 languages. Figure 1 demonstrates the growth rate (6.46% per year) of publications in this field.

**Citation Profile of Articles**

**Fig.2:**
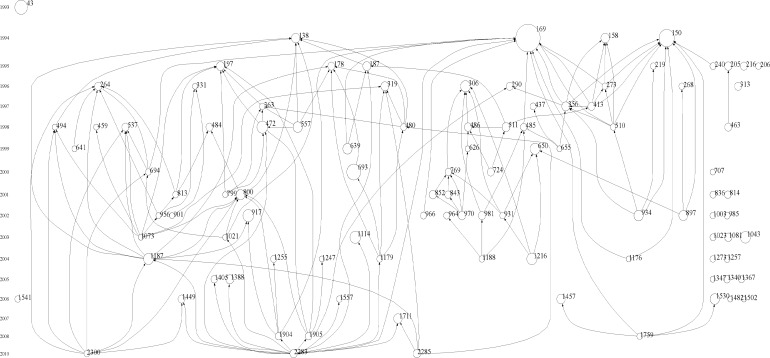
Histogram map of 20 years of research in DR

**Figure 3:**
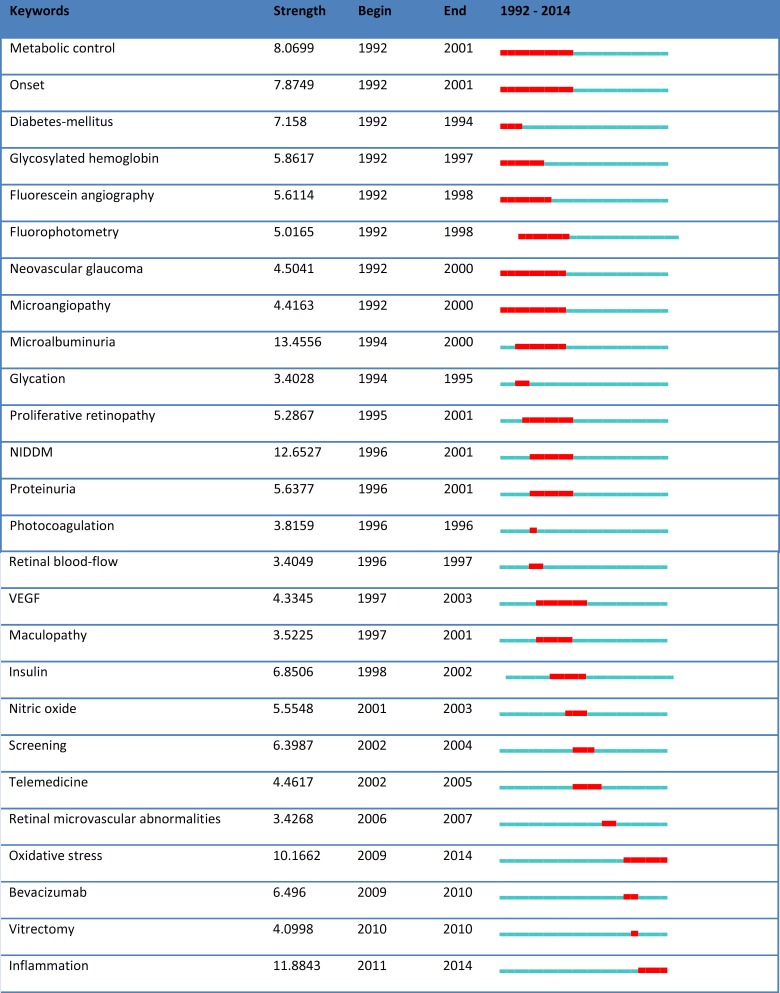
Keywords with the largest citation burst and the corresponding years

The total LCS citations were 12,830 times and the GCS citations were 62,327 times. The average citation per paper (C/P) was 19.31. Table 1 shows the articles that were cited ≥ 100. Appendix 1 shows the highly cited articles in this field. Figure 2 shows the histogram map of 20 years of research in this field. Keywords that generated citation bursts during this period were as follows: Metabolic control, Onset, Diabetes-mellitus, Glycosylated hemoglobin, Fluorescein angiography, Fluorophotometry, Neovascular glaucoma, Microangiopathy, Microalbuminuria, Glycation, Proliferative retinopathy, NIDDM, Proteinuria, Photocoagulation, Retinal blood-flow, VEGF, Maculopathy, Insulin, Nitric oxide, Screening, Telemedicine, Retinal microvascular abnormalities, Oxidative stress, Bevacizumab, Vitrectomy, and inflammation (Fig. 3). 

**Subject Analysis and Publisher of Documents**

The most frequent topics of the top 10 highly cited papers were translational research (30%) and epidemiologic studies (70%) (Table1).

**Profiles of Most Influential Authors and Journals**

The highest number of articles was published by Dr. R. Klein with 133 articles (Table 2). When analyzed by the number of papers in DR, 14 of the top 20 journals were ophthalmology journals and the remaining were diabetes journals. However, when using the same calculation based on the citation number (TLCS), 6 journals were diabetes journals, 12 journals were ophthalmology journals, and 2 journals were general subject medicine journals. When analyzed by the TGCS, highly cited papers were published in ophthalmology journals, diabetes journals, general medicine journals, neurology journals and pathology journals (Table 3). Most DR articles were in English (3,058 articles) followed by German (54 articles), French (47 articles), and Spanish (21 articles). Articles were written in a total of 10 languages (English, German, French, Spanish, Portuguese, Russian, Chinese, Serbo-Croatian, Slovene, and Turkish).

**Geographical Distribution**

Most of the top 10 Universities and institutions on the list are from the United States and Australia. The first two institutions are the University of Wisconsin and University of Melbourne, based on the number of documents, and the University of Wisconsin and Harvard University in based on citations (Table 4).

In general, 93 countries promoted the field of DR by publishing articles. The United States, United Kingdom, and Japan had the highest number of documents, but the United States, United Kingdom, and Australia had the highest number of citations to their research papers in the field of DR (Table 5).

**Table 1:** Articles With Highest Number of Citations (LCS)

**#**	**Author/ Title / Journal**	**CITATION**
**1**	Aiello Lp, Avery Rl, Arrigg Pg, Keyt Ba, Jampel Hd, Et Al. Vascular Endothelial Growth-Factor In Ocular Fluid Of Patients With Diabetic-Retinopathy And Other Retinal Disorders New England Journal Of Medicine. 1994 Dec 1; 331 (22): 1480-1487	1,877
**2**	Adamis Ap, Miller Jw, Bernal Mt, Damico Dj, Folkman J, Et Al. Increased Vascular Endothelial Growth-Factor Levels In The Vitreous Of Eyes With Proliferative Diabetic-Retinopathy American Journal Of Ophthalmology. 1994 Oct; 118 (4): 445-450	745
**3**	Shannon H, Duffy H, Dahms W, Mayer L, Brillion D, Et Al. Retinopathy And Nephropathy In Patients With Type 1 Diabetes Four Years After A Trial Of Intensive Therapy. New England Journal Of Medicine. 2000 Feb 10; 342 (6): 381-389	622
**4**	Dyck Pj, Kratz Km, Karnes Jl, Litchy Wj, Klein R, Et Al. The Prevalence By Staged Severity Of Various Types Of Diabetic Neuropathy, Retinopathy, And Nephropathy In A Population-Based Cohort - The Rochester Diabetic Neuropathy Study Neurology. 1993 Apr; 43 (4): 817-824	518
**5**	Chaturvedi N, Sjolie Ak, Stephenson Jm, Abrahamian H, Keipes M, Et Al. Effect Of Lisinopril On Progression Of Retinopathy In Normotensive People With Type 1 Diabetes Lancet. 1998 Jan 3; 351 (9095): 28-31	393
**6**	Wilkinson Cp, Ferris Fl, Klein Re, Lee Pp, Agardh Cd, Et Al. Proposed International Clinical Diabetic Retinopathy And Diabetic Macular Edema Disease Severity Scales Ophthalmology. 2003 Sep; 110 (9): 1677-1682	372
**7**	Schrier Rw, Estacio Ro, Esler A, Mehler P Effects Of Aggressive Blood Pressure Control In Normotensive Type 2 Diabetic Patients On Albuminuria, Retinopathy And Strokes Kidney International. 2002 Mar; 61 (3): 1086-1097	367
**8**	Joussen Am, Poulaki V, Le Ml, Koizumi K, Esser C, Et Al. A Central Role For Inflammation In The Pathogenesis Of Diabetic Retinopathy Faseb Journal. 2004 Jul; 18 (10): 1450-+	358
**9**	Hammes Hp, Du Xl, Edelstein D, Taguchi T, Matsumura T, Et Al. Benfotiamine Blocks Three Major Pathways Of Hyperglycemic Damage And Prevents Experimental Diabetic Retinopathy Nature Medicine. 2003 Mar; 9 (3): 294-299	343
**10**	Miyamoto K, Khosrof S, Bursell Se, Rohan R, Murata T, Et Al. Prevention Of Leukostasis And Vascular Leakage In Streptozotocin-Induced Diabetic Retinopathy Via Intercellular Adhesion Molecule-1 Inhibition Proceedings Of The National Academy Of Sciences Of The United States Of America. 1999 Sep 14; 96 (19): 10836-10841	329

## DISCUSSION

We analyzed the subject of highly cited papers, divided them into broad categories of clinical\translational versus basic science research (Appendix 1). Most highly cited papers are epidemiologic or translational science reports. Despite the enormous impact of DR on the quality of life and emotional status of patients, few articles among these highly cited papers addressed this subject. Highly cited reports were also addressing the following topics more frequently: laser photocoagulation and angiogenesis. As Appendix 2 shows, there is a recent trend toward more translational research such as biomarker discovery. Areas such as gene therapy and micro-RNA are among the recent hot topics. Citation burst analysis showed that certain topics are very popular such as the role of inflammation or oxidative stress in the pathogenesis of DR. In general, in the field of ophthalmology, there was an increase in the proportion of articles related to medical retina, compared to other subspecialties, between 2005 and 2009. In an analytical study of the ophthalmology research papers, case-control or cohort studies comprised most study designs (40.1%), followed by nonanalytic studies (28.7%), basic science (24.6%), randomized controlled trials (RCTs) (3.3%), review articles (2.6%), and meta-analyses (0.3%) (12). However, this was not the trend in diabetes retinopathy research. The term “citation analysis” covers concepts such as journal impact factor (JIF), the immediacy index, and cited and citing half-lives. The results of citation analysis should be interpreted concurrently with the results of the JIF because ranking of research groups on the basis of JIF has little correlation to a ranking of the same groups on the basis of citation frequency.

**Table 2:** The Most Active Authors in the Field of DR Research

**#**	**Author**	**Recs**	**TLCS**	**TGCS**
**1**	Klein R	133	1653	6084
**2**	Wong Ty	76	595	1868
**3**	Klein Bek	69	861	2933
**4**	Wang Jj	50	455	1504
**5**	Moss Se	44	717	2642
**6**	Sharma T	37	163	352
**7**	Aiello Lp	35	506	3350
**8**	Hammes Hp	35	164	1616
**9**	Kowluru Ra	34	276	1063
**10**	Raman R	33	121	230

Thus, authors who are frequently cited but choose to publish in an appropriate but lower JIF-ranked journal would not receive the best evaluation from the institutional Journal Citation Report-based assessment of an author. Overall, in our study, there was no significant correlation between the JIFs and the citation frequency of articles. This can result from several factors; for example, journals with advance online publication had higher impact factors than journals without advance online publication. Thus, factors other than the quality of papers may affect the citation frequency of a paper (13). In a survey of 46 ophthalmology journals to identify the most frequently cited articles using the Science Citation Index Expanded (1975–2006), the 100 most cited articles were published in 13 journals, the utmost articles were in the Archives of Ophthalmology (n = 30), followed by Ophthalmology (n = 27). American Journal of Ophthalmology (n = 11) was in third place. The published articles originated from 10 countries, led by the United States (n = 86) (14). Laser photocoagulation to treat DR was one of the major topics among the 100 most cited articles. In addition, we found that the h-index of DR was 98, which indicates the appreciation of the context of DR within vision research. Publications of Dr. Klein, who is the most active scientist in the field of DR research, are also among the top 100 most cited articles in the field of ophthalmology, which shows the importance of this field. Our results for the field of citation analysis showed that most citation clusters were generated by few countries and few journals, mostly from the United States and Australian institutions. This fact may be because of the overwhelming influence of the United States on research. However, it may also be because of a tendency for American authors to cite local papers and for authors in other parts of the world to publish in and cite American journals (15).

**Table 3:** Journals with Highest Number of Papers in This Field

#	Journal	Records	Citation	2 year IF	5 year IF
1	Investigative Ophthalmology & Visual Science	167	4378	3.441	3.730
2	Diabetes Care	162	4463	7.735	7.555
3	Diabetic Medicine	124	2380	3.241	3.303
4	British Journal Of Ophthalmology	115	2845	2.725	3.023
5	Ophthalmology	103	4349	5.563	5.777
6	Retina-The Journal Of Retinal And Vitreous Diseases	98	1482	2.825	2.761
7	Diabetes Research And Clinical Practice	97	898	2.741	2.618
8	American Journal Of Ophthalmology	89	3275	3.631	4.292
9	Diabetologia	87	2968	6.487	6.772
10	Eye	83	1124	1.818	1.883
11	Graefes Archive For Clinical And Experimental Ophthalmology	82	1067	1.932	2.037
12	Archives Of Ophthalmology	81	3787	3.826	4.160
13	Diabetes	77	4552	7.895	8.611
14	Journal Of Diabetes And Its Complications	55	649	2.056	2.076
15	Molecular Vision	52	499	1.987	2.311
16	Ophthalmologica	48	427	1.412	1.236
17	Acta Ophthalmologica Scandinavica	47	456	-	-
18	Clinical And Experimental Ophthalmology	45	324	1.964	2.047
19	Acta Ophthalmologica	43	379	2.345	2.428
20	Current Eye Research	36	452	1.710	1.702

**Table 4:** Institutions with highest number of papers

#	**Institution**	**Records**	**TLCS**	**TGCS**
**1**	University Wisconsin	168	2020	7248
**2**	University Melbourne	106	884	2721
**3**	Harvard University	80	938	6029
**4**	University Sydney	75	631	2205
**5**	Natl University Singapore	64	501	1433
**6**	Wayne State University	53	331	1584
**7**	Johns Hopkins University	43	320	1160
**8**	Northeastern Illinois University	39	522	1921
**9**	Case Western Reserve University	36	328	2064
**10**	Aarhus University Hospital	31	240	976
**11**	The University of Tokyo	31	63	312
**12**	Singapore National Eye Center	30	149	345
**13**	Joslin Diabetes Center	29	413	2911
**14**	Shanghai Jiao Tong University	29	46	221
**15**	Capital Med University	28	94	204
**16**	University Heidelberg	28	112	1152
**17**	Sankara Nethralaya	25	92	179
**19**	University Oklahoma	25	130	654
**20**	St Thomas Hospital	24	301	1102

**Table 5:** Countries With Highest Number of Publications in the Field of DR

**#**	**Country**	**Recs**	TLCS	TGCS
**1**	USA	837	5682	29687
**2**	UK	348	1948	7952
**3**	Japan	330	1051	6373
**4**	Peoples R China	220	386	1557
**5**	Australia	206	1210	4050
**6**	Germany	184	736	4974
**7**	Italy	123	348	1629
**8**	India	115	477	1319
**9**	Spain	100	219	1218
**10**	Denmark	92	515	2226
**11**	France	90	298	1476
**12**	Singapore	83	551	1584
**13**	Brazil	76	204	837
**14**	Sweden	75	318	1591
**15**	Turkey	68	132	634
**16**	Canada	66	221	1163
**17**	South Korea	66	118	517

Factors that influence the number of citations that can be obtained by a scientific paper include (1) the merit of journal of publication and (2) the number of references that citing papers use, which is substantially affected by the differences between fields. Also, (3) the number of scientists active in the same field or subfield is important when there are relatively few colleagues working on the same topic. Thus, if for example, more scientists are working on the laser treatment of DR, then there would be heterogeneity between subfields. This may account for the difference in the number of citations between the various types of research papers in DR. For example, scientists active in more basic fields can obtain different numbers of citations than more clinically oriented scientists (15). Among the top 100 cited articles, we could determine that scientists active in the field of translational research and those who were authors on epidemiological studies and RCTs could receive significantly more citations. Much of the burden of visual disorders could be alleviated through at least the three routes: prevention and diagnostic screening, medical treatment of diagnosed conditions, and rehabilitation and support services for those with visual impairment.

Each year, tens of thousands of articles in these areas are published that discuss the medical, policy, and economic aspects of visual problems. Despite this excellent and growing body of work, several areas of research remain virtually nonexistent such as comparing the population benefits of investments in medical treatments for people with vision-threatening disease, compared to rehabilitation and adaptive services for people who have previously acquired impairment. To provide better guidelines for vision research, five major priorities for research were determined by four authorities in A Vision for Horizon 2020. These priorities included neuron–glia interaction, gene therapy in retinal diseases, micro-incision cataract surgery, and femtosecond laser surgery. Improving care and care delivery in the Third World countries has also been mentioned as a research priority. The experts felt that these priority settings may be biased since they are significantly different from topics set by other authorities (16). The results of our and similar studies would help to more accurately determine research priorities in the field of DR. In conclusion, this report is the first scientometric analysis of the field of DR and can be a roadmap for future research policy in this important field. 

In conclusion, this report as the first scientometric analysis of the field of DR, can be regarded as roadmap for future research policy making in this important field.

**Appendix-1:** Top most cited articles in the past 20 years.

**#**	**Author / Title/ Journal**	**Citation**
**1**	169 Aiello LP, Avery Rl, Arrigg PG, Keyt BA, Jampel HD, et al. Vascular Endothelial Growth-Factor In Ocular Fluid Of Patients With Diabetic-Retinopathy And Other Retinal Disorders New England Journal Of Medicine. 1994 Dec 1; 331 (22): 1480-1487	1877
**2**	150 Adamis AP, Miller JW, Bernal MT, Damico DJ, Folkman J, et al. Increased Vascular Endothelial Growth-Factor Levels In The Vitreous Of Eyes With Proliferative Diabetic-Retinopathy American Journal Of Ophthalmology. 1994 OCT; 118 (4): 445-450	745
**3**	693 Shannon H, Duffy H, Dahms W, Mayer L, Brillion D, et al. Retinopathy and nephropathy in patients with type 1 diabetes four years after a trial of intensive therapy. NEW ENGLAND JOURNAL OF MEDICINE. 2000 FEB 10; 342 (6): 381-389	622
**4**	43 Dyck Pj, Kratz Km, Karnes Jl, Litchy Wj, Klein R, et al. The Prevalence By Staged Severity Of Various Types Of Diabetic Neuropathy, Retinopathy, And Nephropathy In A Population-Based Cohort - The Rochester Diabetic Neuropathy Study NEUROLOGY. 1993 APR; 43 (4): 817-824	518
**5**	472 Chaturvedi N, Sjolie AK, Stephenson JM, Abrahamian H, Keipes M, et al. Effect of lisinopril on progression of retinopathy in normotensive people with type 1 diabetes LANCET. 1998 JAN 3; 351 (9095): 28-31	393
**6**	1114 Wilkinson CP, Ferris FL, Klein RE, Lee PP, Agardh CD, et al. Proposed international clinical diabetic retinopathy and diabetic macular edema disease severity scales Ophthalmology. 2003 SEP; 110 (9): 1677-1682	372
**7**	917 Schrier RW, Estacio RO, Esler A, Mehler P Effects of aggressive blood pressure control in normotensive type 2 diabetic patients on albuminuria, retinopathy and strokes KIDNEY INTERNATIONAL. 2002 MAR; 61 (3): 1086-1097	367
**8**	1216 Joussen AM, Poulaki V, Le ML, Koizumi K, Esser C, et al. A central role for inflammation in the pathogenesis of diabetic retinopathy FASEB JOURNAL. 2004 JUL; 18 (10): 1450-+	358
**9**	1043 Hammes HP, Du XL, Edelstein D, Taguchi T, Matsumura T, et al. Benfotiamine blocks three major pathways of hyperglycemic damage and prevents experimental diabetic retinopathy Nature Medicine. 2003 MAR; 9 (3): 294-299	343
**10**	650 Miyamoto K, Khosrof S, Bursell SE, Rohan R, Murata T, et al. Prevention of leukostasis and vascular leakage in streptozotocin-induced diabetic retinopathy via intercellular adhesion molecule-1 inhibition Proceedings Of The National Academy Of Sciences Of The United States Of America. 1999 SEP 14; 96 (19): 10836-10841	329
**11**	306 Mizutani M, Kern TS, Lorenzi M Accelerated death of retinal microvascular cells in human and experimental diabetic retinopathy Journal Of Clinical Investigation. 1996 JUN 15; 97 (12): 2883-2890	322
**12**	1187 Kempen JH, O'Colmam BJ, Leske C, Haffner SM, Klein R, et al. The prevalence of diabetic retinopathy among adults in the United States ARCHIVES OF OPHTHALMOLOGY. 2004 APR; 122 (4): 552-563	322
**13**	639 Antonetti DA, Barber AJ, Hollinger LA, Wolpert EB, Gardner TW Vascular endothelial growth factor induces rapid phosphorylation of tight junction proteins occludin and zonula occluden 1 - A potential mechanism for vascular permeability in diabetic retinopathy and tumors JOURNAL OF BIOLOGICAL CHEMISTRY. 1999 AUG 13; 274 (33): 23463-23467	290
**14**	1530 Avery RL, Pearlman J, Pieramici DJ, Rabena MD, Castellarin AA, et al. Intravitreal bevacizumab (Avastin) in the treatment of proliferative diabetic retinopathy OPHTHALMOLOGY. 2006 OCT; 113 (10): 1695-1705	288
**15**	934 Awata T, Inoue K, Kurihara S, Ohkubo T, Watanabe M, et al. A common polymorphism in the 5 '-untranslated region of the VEGF gene is associated with diabetic retinopathy in type 2 diabetes DIABETES. 2002 MAY; 51 (5): 1635-1639	286
**16**	557 Klein R, Klein BEK, Moss SE, Cruickshanks KJ The Wisconsin epidemiologic study of diabetic retinopathy: XVII - The 14-year incidence and progression of diabetic retinopathy and associated risk factors in type 1 diabetes OPHTHALMOLOGY. 1998 OCT; 105 (10): 1801-1815	279
**17**	800 Stratton IM, Kohner EM, Aldington SJ, Turner RC, Holman RR, et al. UKPDS 50: Risk factors for incidence and progression of retinopathy in Type II diabetes over 6 years from diagnosis DIABETOLOGIA. 2001 FEB; 44 (2): 156-163	273
**18**	897 Joussen AM, Poulaki V, Mitsiades N, Kirchhof B, Koizumi K, et al. Nonsteroidal anti-inflammatory drugs prevent early diabetic retinopathy via TNF-alpha suppression FASEB JOURNAL. 2002 JAN; 16 (1): 438-+	257
**19**	158 Malecaze F, Clamens S, Simorrepinatel V, Mathis A, Chollet P, et al. Detection Of Vascular Endothelial Growth-Factor Messenger-Rna And Vascular Endothelial Growth Factor-Like Activity In Proliferative Diabetic-Retinopathy ARCHIVES OF OPHTHALMOLOGY. 1994 NOV; 112 (11): 1476-1482	247
**20**	138 Klein R, Klein Bek, Moss Se, Cruickshanks Kj The Wisconsin Epidemiologic-Study of Diabetic-Retinopathy .14. 10-Year Incidence and Progression of Diabetic-Retinopathy ARCHIVES OF OPHTHALMOLOGY. 1994 SEP; 112 (9): 1217-1228	244
**21**	1457 Spaide RF, Fisher YL Intravitreal bevacizumab (Avastin) treatment of proliferative diabetic retinopathy complicated by vitreous hemorrhage Retina-The journal Of Retinal And Vitreous Diseases. 2006 Mar; 26 (3): 275-278	242
**22**	1711 Keech AC, Mitchell P, Summonen PA, O'Day J, Davis TME, et al. Effect of fenofibrate on the Need for Laser Treatment for Diabetic Retinopathy (FIELD study): a Randomized Controlled Trial LANCET. 2007 NOV 17; 370 (9600): 1687-1697	229
**23**	356 Amin RH, Frank RN, Kennedy A, Eliott D, Puklin JE, et al. Vascular endothelial growth factor is present in glial cells of the retina and optic nerve of human subjects with nonproliferative diabetic retinopathy investigative Ophthalmology & Visual Science. 1997 Jan; 38 (1): 36-47	222
**24**	319 Chew EY, Klein ML, Ferris FL, Remaley NA, Murphy RP, et al. Association of elevated serum lipid levels with retinal hard exudate in diabetic retinopathy - Early treatment diabetic retinopathy study (ETDRS) report 22 Archives Of Ophthalmology. 1996 SEP; 114 (9): 1079-1084	220
**25**	1388 Watanabe D, Suzuma K, Matsui S, Kurimoto M, Kiryu J, et al. Erythropoietin as a retinal angiogenic factor in proliferative diabetic retinopathy New England Journal Of Medicine. 2005 AUG 25; 353 (8): 782-792	220
**26**	197 Aldington SJ, Kohner Em, Meuer S, Klein R, Sjolie Ak Methodology For Retinal Photography And Assessment Of Diabetic-Retinopathy - The Eurodiab Iddm Complications StuDY Diabetologia. 1995 Apr; 38 (4): 437-444	219
**27**	511 Lieth E, Barber AJ, Xu BY, Dice C, Ratz MJ, et al. Glial reactivity and impaired glutamate metabolism in short-term experimental diabetic retinopathy DIABETES. 1998 May; 47 (5): 815-820	219
**28**	187 Klein R, Klein Bek, Moss Se, Cruickshanks Kj The Wisconsin Epidemiologic-Study of Diabetic-Retinopathy .15. The Long-Term Incidence of Macular Edema OPHTHALMOLOGY. 1995 JAN; 102 (1): 7-16	211
**29**	2283 Cheung N, Mitchell P, Wong TY Diabetic retinopathy LANCET. 2010 JUL 10; 376 (9735): 124-136	199
**30**	970 Stitt A, Gardiner TA, Anderson NL, Canning P, Frizzell N, et al. The AGE inhibitor pyridoxamine inhibits development of retinopathy in experimental diabetes DIABETES. 2002 SEP; 51 (9): 2826-2832	196
**31**	852 Kowluru RA, Tang J, Kern TS Abnormalities of retinal metabolism in diabetes and experimental galactosemia VII. Effect of long-term administration of antioxidants on the development of retinopathy Diabetes. 2001 AUG; 50 (8): 1938-1942	193
**32**	1081 Kramer HJ, Nguyen QD, Curhan G, Hsu CY Renal insufficiency in the absence of albuminuria and retinopathy among adults with type 2 diabetes mellitus Jama-Journal Of The American Medical Association. 2003 JUN 25; 289 (24): 3273-3277	193
**33**	2285 Chew EY, Ambrosius WT, Davis MD, Danis RP, Gangaputra S, et al. Effects of Medical Therapies on Retinopathy Progression in Type 2 Diabetes. New England Journal Of Medicine. 2010 JUL 15; 363 (3): 233-244	186
**34**	486 Mizutani M, Gerhardinger C, Lorenzi M Muller cell changes in human diabetic retinopathy DIABETES. 1998 MAR; 47 (3): 445-449	185
**35**	205 Tarnow L, Cambien F, Rossing P, Nielsen Fs, Hansen Bv, et al. Lack Of Relationship Between An Insertion Deletion Polymorphism In The Angiotensin I-Converting Enzyme Gene And Diabetic Nephropathy And Proliferative Retinopathy In Iddm Patients DIABETES. 1995 MAY; 44 (5): 489-494	181
**36**	463 Fujisawa T, Ikegami H, Kawaguchi Y, Hamada Y, Ueda H, et al. Meta-analysis of association of insertion/deletion polymorphism of angiotensin I-converting enzyme gene with diabetic nephropathy and retinopathy DIABETOLOGIA. 1998 JAN; 41 (1): 47-53	179
**37**	724 Rungger-Brandle E, Dosso AA, Leuenberger PM Glial reactivity, an early feature of diabetic retinopathy Investigative Ophthalmology & Visual Science. 2000 JUN; 41 (7): 1971-1980	179
**38**	178 Shamoon H, Duffy H, Fleischer N, Engel S, Saenger P, et al. The Effect Of Intensive Diabetes Treatment On The Progression Of Diabetic-Retinopathy In Insulin-Dependent Diabetes-Mellitus - The Diabetes Control And Complications Trial Archives Of Ophthalmology. 1995 Jan; 113 (1): 36-51	176
**39**	216 Kohner Em, Patel V, Rassam Smb Role Of Blood-Flow And Impaired Autoregulation In The Pathogenesis Of Diabetic-Retinopathy DIABETES. 1995 Jun; 44 (6): 603-607	174
**40**	484 Kohner EM, Aldington SJ, Stratton IM, Manley SE, Holman RR, et al. United kingdom prospective diabetes study, 30 - Diabetic retinopathy at diagnosis of non-insulin-dependent diabetes mellitus ann associated risk factors Archives Of Ophthalmology. 1998 MAR; 116 (3): 297-303	174
**41**	981 Hammes HP, Lin JH, Renner O, Shani M, Lundqvist A, et al. Pericytes and the pathogenesis of diabetic retinopathy DIABETES. 2002 OCT; 51 (10): 3107-3112	174
**42**	537 Harris MI, Klein R, Cowie CC, Rowland M, Byrd-Holt DD Is the risk of diabetic retinopathy greater in non-Hispanic blacks and Mexican Americans than in non-Hispanic whites with type 2 diabetes? A US population study DIABETES CARE. 1998 AUG; 21 (8): 1230-1235	170
**43**	264 Javitt JC, Aiello LP Cost-effectiveness of detecting and treating diabetic retinopathy ANNALS OF INTERNAL MEDICINE. 1996 JAN 1; 124 (1): 164-169	167
**44**	1449 Wong TY, Klein R, Islam A, Frances M, Folsom AR, et al. Diabetic retinopathy in a multi-ethnic cohort in the United States American Journal Of Ophthalmology. 2006 MAR; 141 (3): 446-455	165
**45**	313 Tachi N, Ogino N Vitrectomy for diffuse macular edema in cases of diabetic retinopathy American Journal Of Ophthalmology. 1996 AUG; 122 (2): 258-260	162
**46**	413 Ambati J, Chalam KV, Chawla DK, DAngio CT, Guillet EG, et al. Elevated gamma-aminobutyric acid, glutamate, and vascular endothelial growth factor levels in the vitreous of patients with proliferative diabetic retinopathy Archives Of Ophthalmology. 1997 SEP; 115 (9): 1161-1166	162
**47**	290 Bursell SE, Clermont AC, Kinsley BT, Simonson DC, Aiello LM, et al. Retinal blood flow changes in patients with insulin-dependent diabetes mellitus and no diabetic retinopathy - A video fluorescein angiography study Investigative Ophthalmology & Visual Science. 1996 APR; 37 (5): 886-897	160
**48**	485 Hammes HP, Lin JH, Bretzel RG, Brownlee M, Breier G Upregulation of the vascular endothelial growth factor vascular endothelial growth factor receptor system in experimental background diabetic retinopathy of the rat DIABETES. 1998 MAR; 47 (3): 401-406	159
**49**	769 Kern TS, Tang J, Mizutani M, Kowluru RA, Nagaraj RH, et al. Response of capillary cell death to aminoguanidine predicts the development of retinopathy: Comparison of diabetes and galactosemia Investigative Ophthalmology & Visual Science. 2000 NOV; 41 (12): 3972-3978	158
**50**	1340 Krady JK, Basu A, Allen CM, Xu YP, LaNoue KF, et al. Minocycline reduces proinflammatory cytokine expression, microglial activation, and caspase-3 activation in a rodent model of diabetic retinopathy DIABETES. 2005 MAY; 54 (5): 1559-1565	158
**51**	1405 Genuth S, Sun WJ, Cleary P, Sell DR, Dahms W, et al. Glycation and carboxymethyllysine levels in skin collagen predict the risk of future 10-year progression of diabetic retinopathy and nephropathy in the diabetes control and complications trial and epidemiology of diabetes interventions and complications participants with type 1 diabetes DIABETES. 2005 NOV; 54 (11): 3103-3111	152
**52**	843 Kern TS, Engerman RL Pharmacological inhibition of diabetic retinopathy - Aminoguanidine and aspirin DIABETES. 2001 JUL; 50 (7): 1636-1642	151
**53**	814 Jonas JB, Hayler JK, Sofker A, Panda-Jonas S Intravitreal injection of crystalline cortisone as adjunctive treatment of proliferative diabetic retinopathy American Journal Of Ophthalmology. 2001 APR; 131 (4): 468-471	144
**54**	1905 Chaturvedi N, Porta M, Klein R, Orchard T, Fuller J, et al. Effect of candesartan on prevention (DIRECT-Prevent 1) and progression (DIRECT-Protect 1) of retinopathy in type 1 diabetes: randomised, placebo-controlled trials LANCET. 2008 OCT 18; 372 (9647): 1394-1402	142
**55**	1904 Sjolie AK, Klein R, Porta M, Orchard T, Fuller J, et al. Effect of candesartan on progression and regression of retinopathy in type 2 diabetes (DIRECT-Protect 2): a randomised placebo-controlled trial LANCET. 2008 OCT 18; 372 (9647): 1385-1393	141
**56**	480 Davis MD, Fisher MR, Gangnon RE, Barton F, Aiello LM, et al. Risk factors for high-risk proliferative diabetic retinopathy and severe visual loss: Early treatment diabetic retinopathy study report #18 Investigative Ophthalmology & Visual Science. 1998 Feb; 39 (2): 233-252	140
**57**	1023 Beck RW, Moke PS, Turpin AH, Ferris FL, Sangiovanni JP, et al. A computerized method of visual acuity testing: Adaptation of the early treatment of diabetic retinopathy study testing protocol American Journal Of Ophthalmology. 2003 FEB; 135 (2): 194-205	139
**58**	964 Enge M, Bjarnegard M, Gerhardt H, Gustafsson E, Kalen M, et al. Endothelium-specific platelet-derived growth factor-B ablation mimics diabetic retinopathy Embo Journal. 2002 AUG 15; 21 (16): 4307-4316	138
**59**	985 Walter T, Klein JC, Massin P, Erginay A A contribution of image processing to the diagnosis of diabetic retinopathy - Detection of exudates in color fundus images of the human retina ieee transactions on medical imaging. 2002 Oct; 21 (10): 1236-1243	136
**60**	437 Palmowski AM, Sutter EE, Bearse MA, Fung W Mapping of retinal function in diabetic retinopathy using the multifocal electroretinogram Investigative Ophthalmology & Visual Science. 1997 NOV; 38 (12): 2586-2596	134
**61**	1255 Matthews DR, Stratton IM, Aldington SJ, Holman RR, Kohner EM Risks of progression of retinopathy and vision loss related to tight blood pressure control in type 2 diabetes mellitus - UKPDS 69 Archives Of Ophthalmology. 2004 NOV; 122 (11): 1631-1640	131
**62**	1257 Zheng L, Szabo C, Kern TS Poly(ADP-ribose) polymerase is involved in the development of diabetic retinopathy via regulation of nuclear factor-kappa B DIABETES. 2004 NOV; 53 (11): 2960-2967	131
**63**	836 Hudson BI, Stickland MH, Futers TS, Grant PJ Effects of novel polymorphisms in the RAGE gene on transcriptional regulation and their association with diabetic retinopathy DIABETES. 2001 JUN; 50 (6): 1505-1511	128
**64**	966 Ogata N, Nishikawa M, Nishimura T, Mitsuma Y, Matsumura M Unbalanced vitreous levels of pigment epithelium-derived factor and vascular endothelial growth factor in diabetic retinopathy American Journal Of Ophthalmology. 2002 SEP; 134 (3): 348-353	125
**65**	1367 Aiello LP The effect of ruboxistaurin on visual loss in patients with moderately severe to very severe nonproliferative diabetic retinopathy initial - Results of the protein kinase C beta inhibitor diabetic retinopathy study (PKC-DRS) multicenter randomized clinical trial DIABETES. 2005 JUL; 54 (7): 2188-2197	125
**66**	240 ELNER SG, ELNER VM, JAFFE GJ, STUART A, KUNKEL SL, et al. Cytokines In Proliferative Diabetic-Retinopathy And Proliferative Vitreoretinopathy Current Eye Research. 1995 NOV; 14 (11): 1045-1053	123
**67**	363 Sjolie AK, Stephenson J, Aldington S, Kohner E, Janka H, et al. Retinopathy and vision loss in insulin dependent diabetes in Europe - The EURODIAB IDDM complications study OPHTHALMOLOGY. 1997 FEB; 104 (2): 252-260	120
**68**	1188 Hammes HP, Lin JH, Wagner P, Feng Y, vom Hagen F, et al. Angiopoietin-2 causes pericyte dropout in the normal retina - Evidence for involvement in diabetic retinopathy DIABETES. 2004 APR; 53 (4): 1104-1110	120
**69**	1273 Kowluru RA, Odenbach S Effect of long-term administration of alpha-lipoic acid on retinal capillary cell death and the development of retinopathy in diabetic rats DIABETES. 2004 DEC; 53 (12): 3233-3238	119
**70**	206 Chew Ey, Mills Jl, Metzger Be, Remaley Na, Jovanovicpeterson L, et al. Metabolic Control And Progression Of Retinopathy - The Diabetes In Early-Pregnancy Study Diabetes Care. 1995 May; 18 (5): 631-637	116
**71**	931 Joussen AM, Poulaki V, Tsujikawa A, Qin WY, Qaum T, et al. Suppression of diabetic retinopathy with angiopoietin-1 American Journal Of PathologY. 2002 MAY; 160 (5): 1683-1693	115
**72**	694 Vijan S, Hofer TP, Hayward RA Cost-utility analysis of screening intervals for diabetic retinopathy in patients with type 2 diabetes mellitus JAMA-journal of the American Medical Association. 2000 FEB 16; 283 (7): 889-896	114
**73**	813 Bursell SE, Cavallerano JD, Cavallerano AA, Clermont AC, Birkmire-Peters D, et al. Stereo nonmydriatic digital-video color retinal imaging compared with early treatment diabetic retinopathy study seven standard field 35-mm stereo color photos for determining level of diabetic retinopathy OPHTHALMOLOGY. 2001 MAR; 108 (3): 572-585	114
**74**	1003 Goebel W, Kretzchmar-Gross T Retinal thickness in diabetic retinopathy - A study using optical coherence tomography (OCT) RETINA-THE JOURNAL OF RETINAL AND VITREOUS DISEASES. 2002 DEC; 22 (6): 759-767	114
**75**	1557 Abraham P, Adelman RA, Alfaro DV, Anand R, Antoszyk A, et al. Effect of ruboxistaurin on visual loss in patients with diabetic retinopathy OPHTHALMOLOGY. 2006 DEC; 113 (12): 2221-2230	114
**76**	1541 Jorge R, Costa RA, Comt DC, Cintra LP, Scott IU Intravitreal bevacizumab (Avastin) for persistent new vessels in diabetic retinopathy (IBEPE Study) RETINA-THE JOURNAL OF RETINAL AND VITREOUS DISEASES. 2006 NOV-DEC; 26 (9): 1006-1013	112
**77**	219 KO BCB, LAM KSL, WAT NMS, CHUNG SSM An (A-C)(N) Dinucleotide Repeat Polymorphic Marker At The 5'-End Of The Aldose Reductase Gene Is Associated With Early-Onset Diabetic-Retinopathy In Niddm Patients DIABETES. 1995 JUL; 44 (7): 727-732	111
**78**	1179 Lyons TJ, Jenkins AJ, Zheng DY, Lackland DT, McGee D, et al. Diabetic retinopathy and serum lipoprotein subclasses in the DCCT/EDIC cohort INVESTIGATIVE OPHTHALMOLOGY & VISUAL SCIENCE. 2004 MAR; 45 (3): 910-918	109
**79**	273 Peer J, Folberg R, Itin A, Gnessin H, Hemo I, et al. Upregulated expression of vascular endothelial growth factor in proliferative diabetic retinopathy BRITISH JOURNAL OF OPHTHALMOLOGY. 1996 MAR; 80 (3): 241-245	108
**80**	1759 Arevalo JF, Maia M, Flynn HW, Saravia M, Avery RL, et al. Tractional retinal detachment following intravitreal bevacizumab (Avastin) in patients with severe proliferative diabetic retinopathy BRITISH JOURNAL OF OPHTHALMOLOGY. 2008 FEB; 92 (2): 213-216	108
**81**	268 Limb GA, Chignell AH, Green W, LeRoy F, Dumonde DC Distribution of TNF alpha and its reactive vascular adhesion molecules in fibrovascular membranes of proliferative diabetic retinopathy BRITISH JOURNAL OF OPHTHALMOLOGY. 1996 FEB; 80 (2): 168-173	106
**82**	510 Boulton M, Foreman D, Williams G, McLeod D VEGF localisation in diabetic retinopathy BRITISH JOURNAL OF OPHTHALMOLOGY. 1998 MAY; 82 (5): 561-568	106
**83**	2300 Zhang XZ, Saaddine JB, Chou CF, Cotch MF, Cheng YJ, et al. Prevalence of Diabetic Retinopathy in the United States, 2005-2008 JAMA-JOURNAL OF THE AMERICAN MEDICAL ASSOCIATION. 2010 AUG 11; 304 (6): 649-656	106
**84**	1347 Giebel SJ, Menicucci G, McGuire PG, Das A Matrix metalloproteinases in early diabetic retinopathy and their role in alteration of the blood-retinal barrier LABORATORY INVESTIGATION. 2005 MAY; 85 (5): 597-607	104
**85**	901 Sinthanayothin C, Boyce JF, Williamson TH, Cook HL, Mensah E, et al. Automated detection of diabetic retinopathy on digital fundus images DIABETIC MEDICINE. 2002 FEB; 19 (2): 105-112	102
**86**	1176 Ray D, Mishra M, Ralph S, Read I, Davies R, et al. Association of the VEGF gene with proliferative diabetic retinopathy but not proteinuria in diabetes DIABETES. 2004 MAR; 53 (3): 861-864	102
**87**	1482 Oshima Y, Sakaguchi H, Gomi F, Tano Y Regression of iris neovascularization after intravitreal injection of bevacizumab in patients with proliferative diabetic retinopathy AMERICAN JOURNAL OF OPHTHALMOLOGY. 2006 JUL; 142 (1): 155-158	102
**88**	331 Gardner GG, Keating D, Williamson TH, Elliott AT Automatic detection of diabetic retinopathy using an artificial neural network: A screening tool BRITISH JOURNAL OF OPHTHALMOLOGY. 1996 NOV; 80 (11): 940-944	101
**89**	626 Hammes HP, Alt A, Niwa T, Clausen JT, Bretzel RG, et al. Differential accumulation of advanced glycation end products in the course of diabetic retinopathy DIABETOLOGIA. 1999 JUN; 42 (6): 728-736	101
**90**	641 Brown MM, Brown GC, Sharma S, Shah G Utility values and diabetic retinopathy AMERICAN JOURNAL OF OPHTHALMOLOGY. 1999 SEP; 128 (3): 324-330	101
**91**	1247 Klein BEK, Klein R, McBride PE, Cruickshanks KJ, Palta M, et al. Cardiovascular disease, mortality, and retinal microvascular characteristics in type 1 diabetes - Wisconsin epidemiologic study of diabetic retinopathy ARCHIVES OF INTERNAL MEDICINE. 2004 SEP 27; 164 (17): 1917-1924	101
**92**	956 Lin DY, Blumenkranz MS, Brothers RJ, Grosvenor DM The sensitivity and specificity of single-field nonmydriatic monochromatic digital fundus photography with remote image interpretation for diabetic retinopathy screening: A comparison with ophthalmoscopy and standardized mydriatic color photography AMERICAN JOURNAL OF OPHTHALMOLOGY. 2002 AUG; 134 (2): 204-213	100
**93**	1073 Tapp RJ, Shaw JE, Harper CA, de Courten MP, Balkau B, et al. The prevalence of and factors associated with diabetic retinopathy in the Australian population DIABETES CARE. 2003 JUN; 26 (6): 1731-1737	100
**94**	655 Fortune B, Schneck ME, Adams AJ Multifocal electroretinogram delays reveal local retinal dysfunction in early diabetic retinopathy INVESTIGATIVE OPHTHALMOLOGY & VISUAL SCIENCE. 1999 OCT; 40 (11): 2638-2651	99
**95**	1021 Younis N, Broadbent DM, Vora JP, Harding SP Incidence of sight-threatening retinopathy in patients with type 2 diabetes in the Liverpool Diabetic Eye Study: a cohort study LANCET. 2003 JAN 18; 361 (9353): 195-200	99
**96**	459 Yu T, Mitchell P, Berry G, Li WN, Wang JJ Retinopathy in older persons without diabetes and its relationship to hypertension ARCHIVES OF OPHTHALMOLOGY. 1998 JAN; 116 (1): 83-89	98
**97**	707 Grant MB, Mames RN, Fitzgerald C, Hazariwala KM, Cooper-DeHoff R, et al. The efficacy of octreotide in the therapy of severe nonproliferative and early proliferative diabetic retinopathy - A randomized controlled study DIABETES CARE. 2000 APR; 23 (4): 504-509	98
**98**	799 Chaturvedi N, Sjoelie AK, Porta M, Aldington SJ, Fuller JH, et al. Markers of insulin resistance are strong risk factors for retinopathy incidence in type 1 diabetes - The EURODIAB Prospective Complications Study DIABETES CARE. 2001 FEB; 24 (2): 284-289	98
**99**	1502 Zhang SX, Wang JJ, Gao GQ, Parke K, Ma JX Pigment epithelium-derived factor downregulates vascular endothelial growth factor (VEGF) expression and inhibits VEGF-VEGF receptor 2 binding in diabetic retinopathy JOURNAL OF MOLECULAR ENDOCRINOLOGY. 2006 AUG; 37 (1): 1-12	98
**100**	494 Mitchell P, Smith W, Wang JJ, Attebo K Prevalence of diabetic retinopathy in an older community - The Blue Mountains Eye Study OPHTHALMOLOGY. 1998 MAR; 105 (3): 406-411	97

The numbers before the article indicate the location of the article on the histogram map.

**Appendix 2:** Top Most Cited Articles (past 3 years)

	**Date / Author / Journal**	**GCS**
**1**	206 Tang J, Kern TS	58
	Inflammation in diabetic retinopathy	
	PROGRESS IN RETINAL AND EYE RESEARCH. 2011 SEP; 30 (5): 343-358	
**2**	328 Yau JWY, Rogers SL, Kawasaki R, Lamoureux EL, Kowalski JW, et al.	56
	Global Prevalence and Major Risk Factors of Diabetic Retinopathy	
	DIABETES CARE. 2012 MAR; 35 (3): 556-564	
**3**	49 Barber AJ, Gardner TW, Abcouwer SF	50
	The Significance of Vascular and Neural Apoptosis to the Pathology of Diabetic Retinopathy	
	INVESTIGATIVE OPHTHALMOLOGY & VISUAL SCIENCE. 2011 FEB; 52 (2): 1156-1163	
**4**	23 Colagiuri S, Lee CMY, Wong TY, Balkau B, Shaw JE, et al.	42
	Glycemic Thresholds for Diabetes-Specific Retinopathy Implications for diagnostic criteria for diabetes	
	DIABETES CARE. 2011 JAN; 34 (1): 145-150	
**5**	81 McArthur K, Feng BA, Wu YX, Chen SL, Chakrabarti S	38
	MicroRNA-200b Regulates Vascular Endothelial Growth Factor-Mediated Alterations in Diabetic Retinopathy	
	DIABETES. 2011 APR; 60 (4): 1314-1323	
**6**	80 Zhong Q, Kowluru RA	31
	Epigenetic Changes in Mitochondrial Superoxide Dismutase in the Retina and the Development of Diabetic Retinopathy	
	DIABETES. 2011 APR; 60 (4): 1304-1313	
